# Is It Time to Genotype Beyond HPV16 and HPV18 for Cervical Cancer Screening?

**DOI:** 10.3389/ijph.2022.1604621

**Published:** 2022-05-12

**Authors:** Brandon Wen Bing Chua, Viva Yan Ma, Jonathan Alcántar-Fernández, Hwee Lin Wee

**Affiliations:** ^1^ Saw Swee Hock School of Public Health, National University of Singapore, Singapore, Singapore; ^2^ Health Economics and Outcomes Research, Becton, Dickinson and Company, Singapore, Singapore; ^3^ Strategic Access, Becton, Dickinson and Company, Singapore, Singapore; ^4^ Innovation and Research Department, Salud Digna A.C., Culiacán, Mexico; ^5^ Department of Pharmacy, Faculty of Science, National University of Singapore, Singapore, Singapore

**Keywords:** cervical cancer screening, HPV genotyping, HPV prevalence, HPV vaccination, HPV screening

**The IJPH series “Young Researcher Editorial” is a training project of the Swiss School of Public Health.**

Cervical cancer was designated a global health priority by the World Health Organization in 2018. Though preventable, cervical cancer is expected to affect 700,000 women and claim 400,000 lives annually by 2030. The human papillomavirus (HPV) is responsible for over 90% of cervical cancers, and 14 high-risk HPV (hrHPV) genotypes have been identified. Of these, HPV16 and HPV18 are involved in 70% of cervical cancers [1]. Marking HPV16/18 for immediate colposcopy is now the cornerstone of many national cervical cancer screening (CCS) programs [2]. Though the other 12 hrHPV genotypes have different prevalence and risk profiles, they are currently identified collectively as a pooled result. Patients with these genotypes are managed as though they are a homogenous group, unlike those identified with HPV16/18. However, new evidence suggests that we should further differentiate the management of patients identified with these 12 hrHPV genotypes.

Detecting hrHPV genotypes beyond HPV16/18 can further stratify patients’ risk and guide their treatment. Across these 12 hrHPV genotypes, the risk of cervical intraepithelial neoplasia grade 3 or worse (CIN3+) is stratified widely [3]. For example, in those with HPV31, the CIN3+ risk is 7.9%–9.8%; in those with HPV33, the CIN3+ risk is 5.4%–15.0%. Since the CIN3+ risk for patients with HPV31 and HPV 33 is similar or higher than those with HPV18 (2.7%–9.0%) [3], immediate colposcopy may also be required. Meanwhile, patients with HPV35/39/51/56/59/66/68 are at low risk for CIN3+ (2.0%) when they have a cervical cytology of low-grade squamous intraepithelial lesion (LSIL) or atypical squamous cells of undetermined significance (ASCUS) [3]. Women with low CIN3+ risk might need only a repeat screening a year later, unless they have a persistent infection that requires colposcopy (an invasive procedure). Persistent infection with the same hrHPV genotype is associated with higher risk of CIN2 and CIN3, than persistent infection with a change in hrHPV genotype (HPV genotype switch) [4].

Identifying additional hrHPV genotypes individually makes it possible to classify and manage patients based on their CIN3+ risk. HPV extended genotyping could be cost-effective in the US because it may reduce colposcopy referrals [5]. In high-resource settings, risk-based screening algorithms may replace screening algorithms based only on HPV16/18, but this may not be feasible in low-resource settings where interventions have yet to be evaluated; more research is required.

Monitoring an extended range of hrHPV genotypes will help us track treatment success of precancerous lesions. During post-treatment surveillance, persistent infections by the same hrHPV genotype can be better differentiated from new infections. Among women who remain HPV-positive after CIN2+ treatment, about half have the same hrHPV genotype [6]. These women should be more closely monitored for possible treatment failure, than those with a HPV genotype switch.

Monitoring additional hrHPV genotypes will provide the evidence base for revising national HPV vaccination policies. Vaccination will provide epidemiological shifts in hrHPV genotypes. Tracking those shifts will provide the evidence required to update guidelines on risk stratification and patient management. Countries with high HPV vaccination coverage can expect HPV16/18 prevalence to decrease, while other hrHPV genotypes will predominate. HPV vaccination has reduced the overall prevalence of HPV16/18 in Australia to 2.1%, but the prevalence of the 12 other hrHPV genotypes remains high (7.1%) [7]. In settings with high vaccination coverage like Australia, using extended genotyping to surveil HPV vaccine and non-vaccine targeted genotypes will help researchers identify subsequent vaccine targets. Countries with poor vaccination coverage should also surveil HPV to establish baseline hrHPV prevalence. Comparisons in hrHPV prevalence can be made between vaccinated and pre-vaccinated women to evaluate the coverage and effectiveness of national vaccination programs [8].

However, in low- and middle-income countries (LMICs), adopting HPV extended genotyping can be challenging. Besides affordability, the capacity to conduct nucleic acid–based tests and quickly deliver results is an issue [9]. A country may need strategic partnerships across public, private and non-governmental sectors to ensure broad adoption of HPV extended genotyping. In Mexico, an upper-middle income economy with large health care access disparities and a fragmented health system [10, 11], partnerships between the medical device industry and a non-governmental organization (NGO) have helped the health system adopt automated screening technologies with low error rates [12, 13]. This includes HPV extended genotyping capacities for HPV surveillance and CCS [14]. Collaborations between the public and the NGO have also expanded CCS in rural and low-income communities, providing access to efficient, high quality and low-cost screening services among underserved populations in Mexico [12, 13]. Automation has made these services financially sustainable from their operational revenue, while facilitating patient access to screening: automated HPV genotyping and cytology is carried out on specimens from over 90 cities, consolidated at a national reference laboratory.

Emerging technologies that expand HPV genotyping beyond HPV16/18 open the doors for developing clinical guidelines that improve cervical cancer outcomes. Expanded genotyping should allow us to stratify patients by genotype-specific risk of precancers or cancers and limit invasive procedures to those who need them. It will also reveal epidemiological trends in the evolution of HPV, providing data required to inform HPV vaccination policies. Though it may be difficult to implement these new technologies in LMICs, some of these barriers may be surmounted if governments can establish strategic partnerships among public, private, and non-governmental sectors.
